# Subclinical Leaflets Thrombosis After Transcatheter Replacement of Bicuspid vs. Tricuspid Aortic Valve

**DOI:** 10.3389/fcvm.2021.790069

**Published:** 2021-12-22

**Authors:** Gangjie Zhu, Jiaqi Fan, Dao Zhou, Hanyi Dai, Qifeng Zhu, Yuxin He, Yuchao Guo, Lihan Wang, Xianbao Liu, Jian'an Wang

**Affiliations:** ^1^Department of Cardiology, Second Affiliated Hospital Zhejiang University School of Medicine, Hangzhou, China; ^2^Zhejiang University School of Medicine, Hangzhou, China

**Keywords:** transcatheter aortic valve replacement, subclinical leaflet thrombosis, bicuspid aortic valve, tricuspid aortic valve, hypoattenuated leaflet thickening

## Abstract

**Background:** Subclinical leaflet thrombosis (SLT) is an important sequela that compromises the durability of the bioprosthetic valve.

**Objectives:** To better determine the effect of SLT in bicuspid aortic valve (BAV), we performed a retrospective assessment of CT-defined SLT in BAV and tricuspid aortic valve (TAV) stenotic patients.

**Methods:** We consecutively collected patients undergoing the TAVR between August 2015 and March 2020 in our center. A total of 170 BAV and 201 TAV cases were enrolled. Multidetector computed tomography was performed within 30 days and at 1-year.

**Results:** Twenty cases in the BAV group and 19 cases in the TAV group had hypoattenuated leaflet thickening (HALT) in 30 days (12.5 vs. 9.9%, *p* = 0.449), and 52 cases in BAV and 61 cases in TAV had the HALT (34.9 vs. 36.7%, *p* = 0.733) at 1-year follow-up. The mean aortic gradient (MAG) and effective orifice areas (EOA) values were comparable between the two groups at 30 days (HALT vs. no HALT; 10.8 ± 4.8 vs. 11.3 ± 6.0, *p* = 0.638; 1.6 ± 0.4 vs. 1.6 ± 0.3, *p* = 0.724), and still, no difference was observed in the MAG at 1-year (11.5 ± 5.6 vs. 10.6 ± 5.1, *p* = 0.164). However, the EOA at 1-year was statistically different between the two groups (1.5 ± 0.3 vs. 1.6 ± 0.4, *p* = 0.004). The multivariate logistic regression analysis demonstrated the anticoagulation and age as independent predictors both in the BAV and TAV groups at 1-year. There was no difference in clinical events between the HALT and no HALT group in relevant to BAV or TAV at 1-year follow-up.

**Conclusions:** The presence of subclinical leaflet thrombosis defined by the CT was comparable between the BAV and TAV in the first year after the TAVR procedure. Age and anticoagulation were the independent predictors of the subclinical leaflet thrombosis at 1 year after the TAVR. There was no difference in relevant clinical events between the BAV and TAV groups at 1-year follow-up.

## Introduction

In elderly patients with symptomatic aortic stenosis (AS), transcatheter aortic valve replacement (TAVR) is a less invasive heart procedure to replace the stenotic valve with a favorable prognosis. As the use of the TAVR for the indication of the AS expands to younger and low-risk patients, the goal of developing the durable bioprosthetic valve has been particularly focused on. Subclinical leaflet thrombosis (SLT) is a critical occurrence that jeopardizes the durability of the bioprosthetic valve. Hypoattenuated leaflet thickening (HALT) and reduced leaflet motion (RELM), as detected by multidetector computed tomography (MDCT) were hallmarks of the subclinical leaflet thrombosis (1–4). The occurrence of the SLT in transcatheter valve replacements is about 10–40% (3–7).

Due to severe and asymmetric calcification in the native aortic valves and the deformation of the bioprosthetic frames after the TAVR, the SLT in the bicuspid aortic valve (BAV) is very concerned (8). At present, there is no study to compare the SLT viewed by computed tomography (CT) and its clinical sequelae and prognosis between the BAV and tricuspid aortic valve (TAV). To better explore the SLT in the BAV, this study aimed to retrospectively assess the SLT defined by the CT in the BAV and TAV stenotic patients.

## Methods

### Study Population

This study was a retrospective observational analysis. We consecutively collected patients undergoing the TAVR between August 2015 and March 2020 in our center. Exclusion criteria: (1) Cases lacking the pre-procedure CT to define aortic valve type including quadricuspid valve; (2) Patients received bioprosthetic implant before the TAVR procedure; (3) Patients with contrast agents contraindicated, allergies, and severe renal dysfunction (estimated glomerular filtration rate of ≤ 30 ml/min); (4) Patients lost to follow up; (5) Patients with incomplete or inconclusive CT series.

The morphological type of aortic valve was classified into BAV (including type 0, type 1, and type 2) or TAV according to the Sievers classification (9). The study was approved by the local Ethical Committee and was in accordance with the principles of the Declaration of Helsinki.

### TAVR Procedure and Antithrombotic Regimen

The TAVR procedures were performed in a hybrid operating room. Unfractionated heparin was used (50–70 U/kg) to maintain an activated clotting time (ACT) of >250 s during all procedures. Adopting general anesthesia or local anesthesia with sedation was decided by anesthetists. Transfemoral or non-transfemoral access was used based on the pre-procedure assessment. The majority of cases were implanted with self-expanding valves, and the rest of the patients were implanted with balloon-expandable or mechanically expanding valves. Post-dilatation was employed based on surgeons' discretion. A large proportion of the patients were prescribed dual antiplatelet therapy (DAPT) following the procedures. Oral anticoagulants (OAC) were recommended if the patients had indications of anticoagulation.

### Echocardiography and Laboratory Tests

Transthoracic echocardiography (TTE) was performed before the TAVR procedure, before discharge, and at 30-day and 1-year follow-up. The mean aortic gradient (MAG), effective orifice area (EOA), left ventricular end-diastolic diameter (LVEDd), left atrium diameter, left ventricular ejection fraction (LVEF), and pulmonary arterial systolic pressure (PASP) were measured by the TTE. The results of the TTE were analyzed by experienced echocardiographers. The levels of the D-dimer and N-terminal pro-brain natriuretic peptide (NT-pro-BNP) were tested at each follow-up visit.

### MDCT Acquisition and Analysis

Cardiac contrast-enhanced ECG-gated multidetector computed tomography (MDCT) was performed using Philips Brilliance iCT 256 (Philips Corporation, Amsterdam, Netherlands) or GE revolution CT (GE Healthcare, Chicago, IL, USA) with collimation of 0.6 or 0.8 mm, 100 or 120 kV for imaging.

Patients routinely underwent MDCT scanning before the procedure, before discharge or at 30 days after implantation (first CT) and at 1-year follow-up (second CT). Full phase CT imaging was acquired and analyzed by using 3mensio workstation (Pie Medical Imaging, Maastricht, Netherlands). Two authors (Dao Zhou and Hanyi Dai) evaluated the CT scans independently and one author (Gangjie Zhu) reviewed the data.

### HALT and RELM

The HALT was evaluated in cardiac diastole. The area and thickness of hypoattenuation were measured in a cross-sectional 2D multiplanar reconstruction (MPR) view and corresponding 2D longitudinal MPR view, respectively. If the HALT was found, the RELM had been evaluated in cardiac systole with the 3D or 4D CT. According to the severity of the leaflet reduced motion, the RELM was graded as mild (<50%), moderate (≥50%, <70%), and severe (≥70%) (10). The moderate and severe RELM were denoted as hypoattenuation affecting motion (HAM) (10).

%RELM = (width of hypoattenuation/ (1/2 diameter of the bioprosthesis in the section)·100%.

### Follow-Up and Clinical Adverse Events

Despite this study was a retrospective analysis, patients who underwent the TAVR procedure, were routinely followed up before discharge, and at 30 days and 1 year after the procedure. Clinical adverse events were defined according to the VARC-3 criteria (11).

### Statistical Analysis

Statistical analysis was performed using the Statistical Package for the Social Sciences version 25.0.0 (International Business Machines Corporation, Armonk, NY, USA) and GraphPad Prism version 6.0 (GraphPad Software, San Diego, CA, USA). Continuous variables are presented as mean ± SD or median [interquartile range (IQR)] and were analyzed by Student's *t*-test or Mann-Whitney *U*-test. Categorical variables are presented as count (percentage). And Pearson's chi-squared test or Fisher exact test were used to analyze the categorical variables. Multivariate logistical regression was used to identify predictors of the HALT, which included co-variables with the *p* < 0.10 in the univariable logistical regression. Statistical significance was defined at the *p* < 0.05 with two-tailed tests.

## Results

### Patients Characteristics

**Figure 1:**
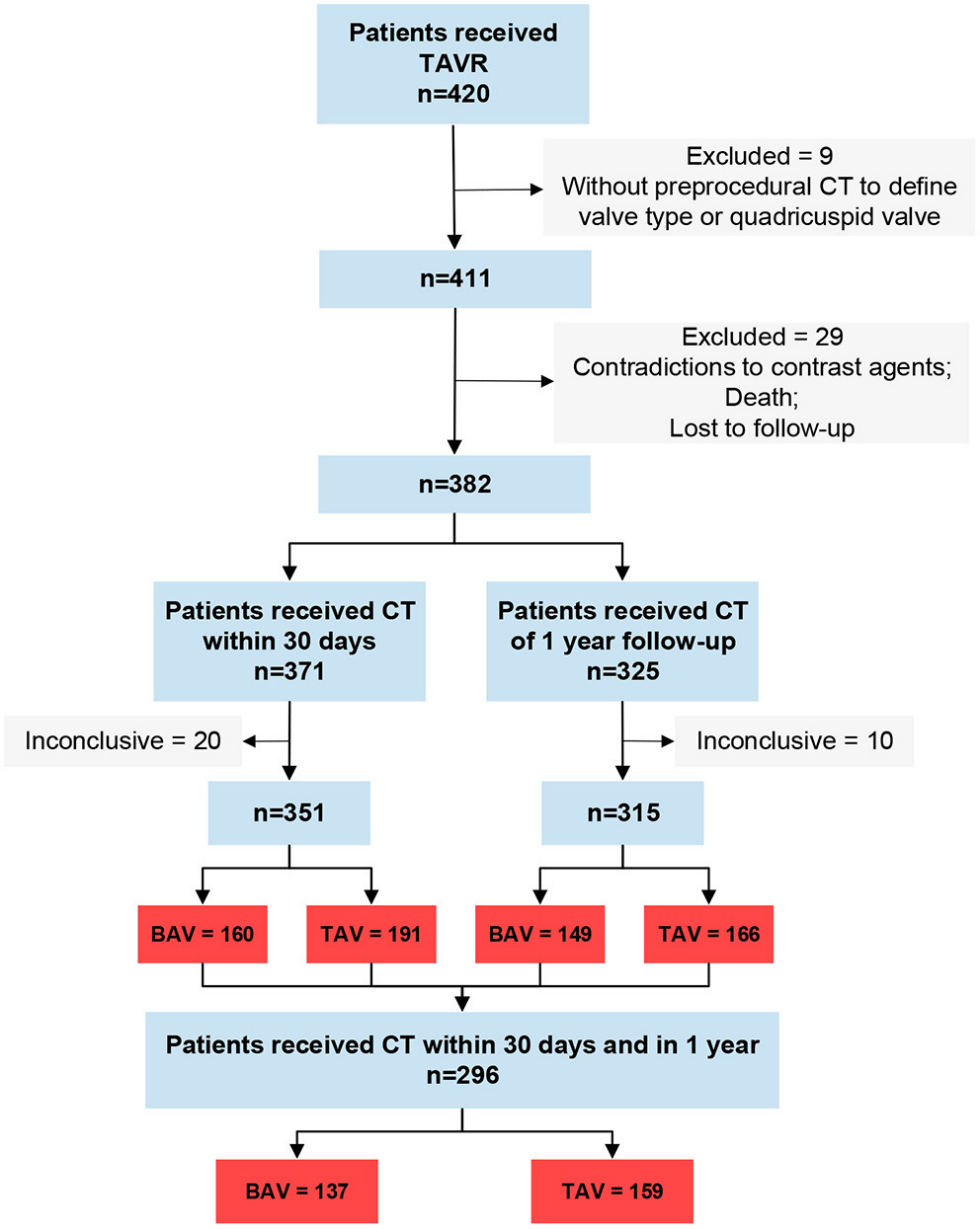
Study flow. Inconclusive, CT can't be analyzed; BAV, icuspid aortic valve; TAV, tricuspid aortic valve.

A total of 420 patients underwent the TAVR procedure between August 2015 and March 2020. Among them, 9 patients were excluded because of lacking pre-procedure CT or having a quadricuspid valve, and 29 patients were excluded because of contradictions to contrast agents, death, or loss to follow-up (Figure 1). A total of 371 patients had CT within 30 days post TAVR procedure, of which 20 CT scans were inconclusive because of poor imaging quality. Three hundred and twenty-five patients received CT at 1-year follow-up but 10 CT scans were inconclusive. Finally, 160 patients with BAV involvement and 191 patients with TAV involvement were included for the first CT (within 30 days post the procedure) images analysis. A total of 149 BAVs and 166 TAVs were included for the second CT (at 1-year follow-up; BAV vs. TAV, 12.3 ± 1.1 vs. 12.6 ± 1.9 months) images analysis. A total of 137 BAVs and 159 TAVs had completed CT scans within 30 days and 1-year follow-up.

Baseline demographic and clinical characteristics are summarized in Table 1. A total of 170 BAV cases and 201 TAV cases were enrolled for this study. Patients in BAV group were younger and had a lower risk than patients in TAV group (age: 75.1 ± 6.6 vs. 76.9 ± 6.7, *p* = 0.013; STS: 6.0 ± 3.7% vs. 7.1 ± 5.1%, *p* = 0.010). Syncope occurred more frequent in the BAV than the TAV (11.8 vs. 6.0%, *p* = 0.048).

**Table 1:** Baseline demographics and clinical characteristics.

	**BAV** ***n =* 170**	**TAV** ***n =* 201**	***P*-value**
Age, yrs	75.1 ± 6.6	76.9 ± 6.7	0.013
Male	97 (57.1)	120 (59.7)	0.607
BMI, kg/m^2^	22.4 ± 3.3	22.6 ± 3.6	0.541
STS PROM, %	6.0 ± 3.7	7.1 ± 5.1	0.010
Smoking	24 (14.1)	32 (15.9)	0.629
Dyslipidemia	29 (17.1)	41 (20.4)	0.413
Hypertension	91 (53.5)	121 (60.2)	0.196
Diabetes mellitus	38 (22.4)	44 (21.9)	0.915
Syncope	20 (11.8)	12 (6.0)	0.048
**NYHA functional class**			
I - II	20 (11.8)	22 (10.9)	0.804
III	84 (49.4)	84 (41.8)	0.142
IV	66 (38.8)	95 (47.3)	0.102
Previous MI	1 (0.6)	5 (2.5)	0.225
Prior PCI	12 (7.1)	23 (11.4)	0.209
Prior CABG	0 (0)	3 (1.5)	0.253
Prior stroke	6 (3.5)	13 (6.5)	0.676
Prior pacemaker	4 (2.4)	5 (2.5)	1.000
Atrial fibrillation/flutter	29 (17.1)	38 (18.9)	0.645
PVD	18 (10.6)	33 (16.4)	0.104
COPD	35 (20.6)	50 (24.9)	0.328
LVEF, %	52.7 ± 14.8	54.9 ± 13.7	0.261

*Values are mean ± SD or number (%)*.

*BMI, body mass index; CABG, coronary artery bypass grafting; COPD, Chronic obstructive pulmonary disease; LVEF, left ventricular ejection fraction; MI, myocardial infarction; NYHA, New York Heart Association; PCI, percutaneous coronary intervention; PROM, Predicted Risk of Mortality; PVD, peripheral vascular diseases; STS, Society of Thoracic Surgeons*.

### TAVR Procedure

Procedural details were listed in Table 2. There was a higher proportion of local anesthesia (90.0 vs. 78.6%, *p* = 0.003), transfemoral access (97.1 vs. 86.6%, *p* < 0.001) and post-dilatation (62.9 vs. 41.8%, *p* < 0.001) in the BAV group. Many BAV patients were implanted with 23–26 mm valve devices compared with the TAV patients (62.9 vs. 41.8%, *p* < 0.001). And a higher percentage of 26–29 mm valve devices were implanted in the patients with the TAV (15.3 vs. 31.8%, *p* < 0.001). No case was converted to surgery of both valves in the BAV and TAV groups.

**Table 2:** Procedural details.

	**BAV** ***n =* 170**	**TAV** ***n =* 201**	***P*-value**
Procedural time, min	71.3 ± 35.2	68.4 ± 41.8	0.494
Local anesthesia	153 (90.0)	158 (78.6)	0.003
**Access**			
Transfemoral	165 (97.1)	174 (86.6)	<0.001
Non-transfemoral	5 (2.9)	27(13.4)	<0.001
**Transcatheter valve type**			
Self-expanding valve	150 (88.2)	164 (81.2)	0.077
Balloon-expandable valve	7 (4.1)	28 (13.9)	0.001
Mechanically expanding valve	13 (7.6)	9 (4.5)	0.198
**Bioprosthetic valve size, mm**			
≤ 23	31 (18.2)	40 (19.9)	0.685
>23, ≤ 26	107 (62.9)	84 (41.8)	<0.001
>26, ≤ 29	26 (15.3)	64 (31.8)	<0.001
> 29	6 (3.5)	13 (6.5)	0.201
Postdilation	107 (62.9)	84 (41.8)	<0.001
Implantation of >1 valve	13 (12.1)	9 (4.5)	0.198
Conversion to surgery	0 (0)	0 (0)	–

*Values are mean ± SD or number (%)*.

### HALT and RELM

**Figure 2:**
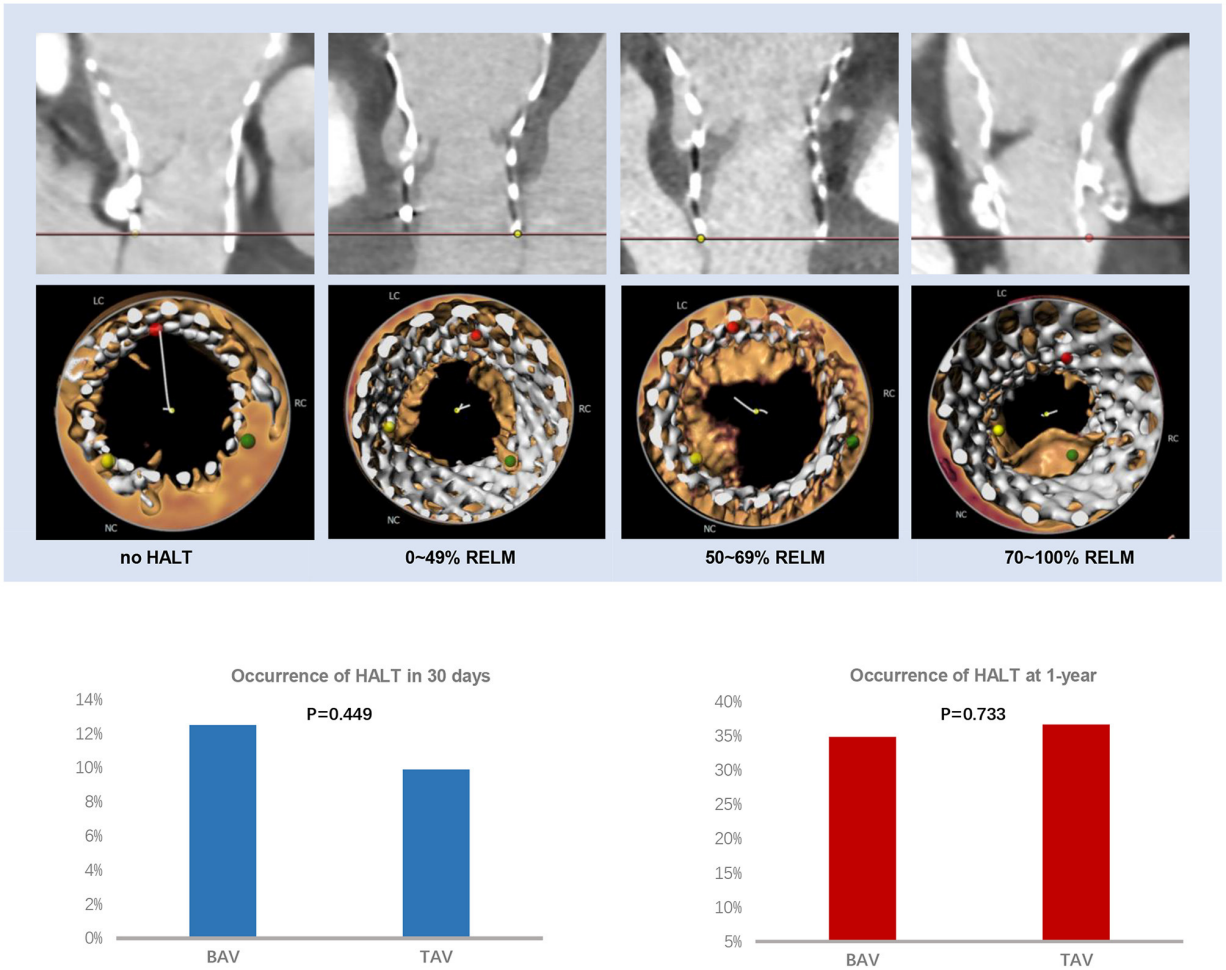
HALT and RELM in BAV and TAV. According to the severity of the leaflet reduced motion, the RELM was graded as mild (<50%), moderate (≥50%, <70%), and severe (≥70%). There was no difference of HALT between the BAV and TAV group within 30 days (12.5 vs. 9.9%, *p* = 0.449) or at 1-year (34.9 vs. 36.7%, *p* = 0.733) follow-up. BAV, bicuspid aortic valve; HALT, hypoattenuated leaflet thickening; RELM, reduced leaflet motion; TAV, tricuspid aortic valve.

A total of 20 cases in the BAV group and 19 cases in the TAV group had HALT in 30 days (12.5 vs. 9.9%, *p* = 0.449) (Table 3; Figure 2). Among them, involvement of one leaflet, two leaflets, and three leaflets were 70.0 vs. 84.2% (*p* = 0.901), 15.0 vs. 10.5% (*p* = 0.663), and 10.0 vs. 5.3% (*p* = 0.335) in the BAV and TAV groups, respectively. The occurrence of the RELM in BAV and TAV was 11.9 and 9.4% (*p* = 0.456) in 30 days. The occurrence of the HAM in BAV and TAV was 5.6 and 2.6% (*p* = 0.152), respectively. Severe RELM was rare in both groups (1.9 vs. 1.0%, *p* = 0.663). Maximal leaflet thickness, maximal area of hypoattenuation, and total area of hypoattenuation were comparable in two groups (Table 3).

**Table 3:** HALT/RELM within 30 days or at 1-year.

	**30 days**			**1-year**		
	**BAV** ***n =* 160**	**TAV** ***n =* 191**	***P*-value**	**BAV** ***n =* 149**	**TAV** ***n =* 166**	***P*-value**
HALT	20 (12.5)	19 (9.9)	0.449	52 (34.9)	61 (36.7)	0.733
One leaflet involved	14 (70.0)	16 (84.2)	0.901	26 (50.0)	38 (62.3)	0.231
Two leaflets involved	3 (15.0)	2 (10.5)	0.663	20 (38.5)	18 (29.5)	0.483
Three leaflets involved	3 (15.0)	1 (5.3)	0.335	6 (11.5)	5 (8.2)	0.624
RELM	19 (11.9)	18 (9.4)	0.456	51 (34.2)	56 (33.7)	0.926
<50%	10 (52.6)	13 (72.2)	0.834	26 (51.0)	34 (60.7)	0.494
≥50%, <70%	6 (30.0)	3 (16.7)	0.310	23 (45.1)	19 (33.9)	0.298
≥70%	3 (15.0)	2 (11.1)	0.663	2 (3.9)	3 (5.4)	1.000
HAM	9 (5.6)	5 (2.6)	0.152	25 (16.8)	22 (13.3)	0.381
Maximal leaflet thickness, mm	3.6 ± 1.9	3.2 ± 2.1	0.513	4.5 ± 2.0	4.0 ± 1.9	0.169
Maximal area of hypoattenuation, mm^2^	42.9 ± 16.2	42.4 ± 21.8	0.935	46.2 ± 19.6	44.9 ± 18.3	0.607
Total area of hypoattenuation, mm^2^	53.6 ± 31.8	47.9 ± 31.9	0.586	71.1 ± 49.3	63.0 ± 37.8	0.229

*Values are mean ± SD or number (%)*.

*BAV, bicuspid aortic valve; HALT, hypoattenuated leaflet thickening; HAM, hypoattenuation affecting motion; RELM, reduced leaflet motion; TAV, tricuspid aortic valve*.

At 1-year follow-up, there were 52 cases in BAV and 61 cases in TAV with HALT (34.9 vs. 36.7%, *p* = 0.733), and 51 cases in BAV and 56 cases in TAV with RELM (34.2 vs. 33.7%, *p* = 0.926) (Table 3; Figure 2). There was no statistical difference with HAM, maximal leaflet thickness, maximal area of hypoattenuation, and total area of hypoattenuation between BAV and TAV.

To eliminate the impact of the device type, we excluded balloon-expandable and mechanically expanding valves. We found the occurrence of HALT was still comparable between the BAV and TAV group within 30 days or at 1 year (BAV vs. TAV, 11.8 vs. 11.6%, *p* = 0.959; 33.3 vs. 34.8%, *p* = 0.800) (Supplementary Table 1A). We also compared the occurrence of HALT in the supra-annular bioprostheses (self-expanding valves) and the inter-annular bioprostheses (balloon-expandable and mechanically expanding valves) group (Supplementary Table 1B). The outcomes were still comparable between the self-expanding valves and the balloon-expandable/mechanically expanding valves groups within 30 days or at 1 year (Supplementary Table 1B).

### HALT/RELM Evolution

**Figure 3:**
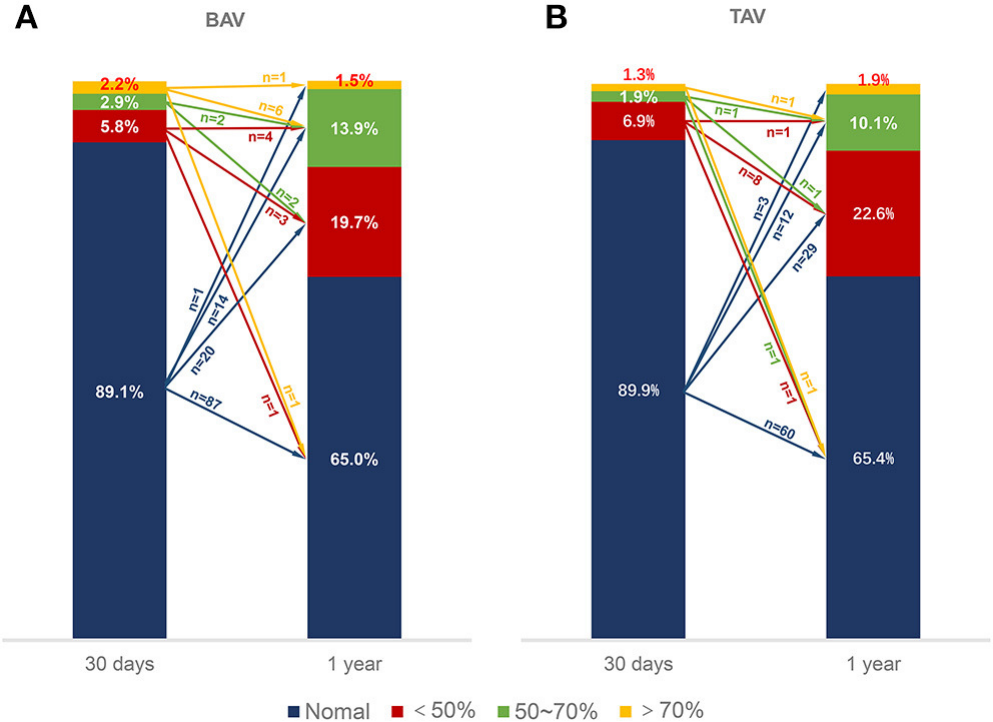
Evolution of RELM. The different color bars represented normal leaflets, <50% RELM, ≥50%, <70% RELM and ≥70% RELM respectively. The different color arrows represented evolution of RELM form 30 days to 1-year follow-up. The numbers upon the color arrows represented the number of patients. BAV, bicuspid aortic valve; RELM, reduced leaflet motion; TAV, tricuspid aortic valve.

A total of 137 patients with BAV and 159 patients with TAV were evaluated for the evolution of HALT/RELM. Fifty cases in the BAV group and 59 cases in the TAV group (36.5 vs. 37.1%, *p* = 0.914) had the evolution of HALT/RELM in 30 days or 1 year (Figure 3). Among them, 5 cases in BAV and 7 cases in TAV regressed; 6 cases in BAV and 8 cases in TAV remained stable; most cases in BAV and TAV (78.0 vs. 74.6%, *p* = 0.879) progressed. Specific data regarding the evolution of HALT/RELM were given in Figure 3; Table 4.

**Table 4:** Regression or progression of RELM.

	**BAV** ***n =* 137**	**TAV** ***n =* 159**	***P*-value**
RELM in 30 days	15 (10.9)	16 (10.1)	0.804
RELM in 1 year	48 (35.0)	55 (34.6)	0.936
RELM in 30 days or 1 year	50 (36.5)	59 (37.1)	0.914
Regression of RELM	5 (10.0)	7 (11.9)	0.743
Progression of RELM	39 (78.0)	44 (74.6)	0.879
No change of RELM	6 (12.0)	8 (13.6)	0.792

*Values are number (%). BAV, bicuspid aortic valve; RELM, reduced leaflet motion; TAV, tricuspid aortic valve*.

### Echocardiographic Valve Assessment

**Figure 4:**
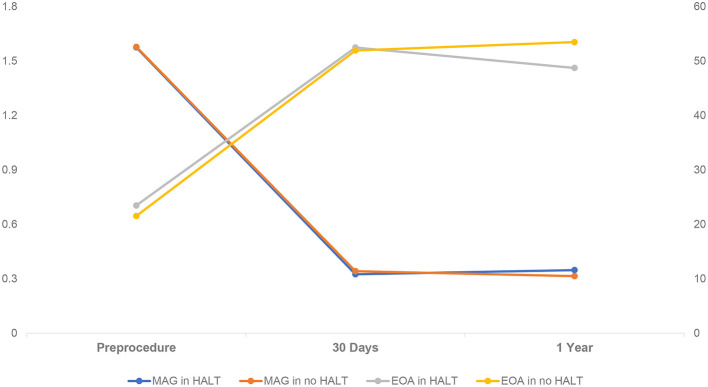
Hemodynamic Change in Patients with HALT or no HALT. There was statistical difference between HALT and no HALT in EOA at 1-year follow-up (1.5 ± 0.3 vs. 1.6 ± 0.4, *p* = 0.004), but not in MAG (11.5 ± 5.6 vs. 10.6 ± 5.1, *p* = 0.164). There was no difference between HALT and no HALT in hemodynamic status at 30 days follow-up. EOA, Effective Orifice Areas; HALT, hypoattenuated leaflet thickening; MAG, mean aortic gradient.

In comparison with the HALT group, the no HALT group had a higher percentage of aortic paravalvular leak of ≥ moderate at 30 days (0 vs. 6.7%) and 1-year (3.5 vs. 11.4%, *p* = 0.006) follow-up (Table 5). The MAG and EOA values were comparable between the two groups at 30 days (HALT vs. no HALT; 10.8 ± 4.8 vs. 11.3 ± 6.0, *p* = 0.638; 1.6 ± 0.4 vs. 1.6 ± 0.3, *p* = 0.724), and still, no difference was observed in the MAG value at 1 year (HALT vs. no HALT; 11.5 ± 5.6 vs. 10.6 ± 5.1, *p* = 0.164) (Table 5; Figure 4). However, the EOA at 1 year was statistically different between the two groups (HALT vs. no HALT; 1.5 ± 0.3 vs. 1.6 ± 0.4, *p* = 0.004). Overall, the hemodynamic status was comparable between the HALT and no HALT group at 30 days, but the HALT group had smaller EOA values at 1 year.

**Table 5:** Transthoracic echocardiography at 30 days and 1-year.

	**30 days**			**1-year**		
	**HALT** ***n =* 39**	**No HALT** ***n =* 312**	***P*-value**	**HALT** ***n =* 149**	**No HALT** ***n =* 201**	***P*-value**
Aortic paravalvular leak ≥ moderate	0 (0)	21 (6.7)	0.148	4 (3.5)	26 (11.4)	0.006
Mean aortic gradient, mmHg	10.8 ± 4.8	11.3 ± 6.0	0.638	11.5 ± 5.6	10.6 ± 5.1	0.164
Effective orifice areas, cm^2^	1.6 ± 0.4	1.6 ± 0.3	0.742	1.5 ± 0.3	1.6 ± 0.4	0.004
Transvalvular regurgitation ≥ moderate	0 (0)	2 (0.6)	1.000	1 (0.9)	1 (0.5)	1.000
LVEDd, mm	4.7 ± 0.7	4.7 ± 0.8	0.662	4.5 ± 0.7	4.6 ± 0.7	0.362
LA, mm	3.9 ± 0.7	4.0 ± 0.6	0.122	4.0 ± 0.7	4.1 ± 0.6	0.127
LVEF, %	57.1 ± 12.1	58.4 ± 10.3	0.471	61.1 ± 9.4	61.8 ± 9.2	0.541
Mitral regurgitation ≥ moderate	2 (5.1)	29 (9.3)	0.554	10 (8.8)	17 (8.5)	0.924
Tricuspid regurgitation ≥ moderate	1 (2.6)	26 (8.3)	0.337	13 (11.4)	21 (10.4)	0.793
PASP, mmHg	30.4 ± 6.8	32.1 ± 9.6	0.382	33.1 ± 9.4	32.6 ± 8.8	0.671

*Values are mean ± SD or number (%)*.

*HALT, hypoattenuated leaflet thickening; LA, left atrium; LVEDd, left ventricular end diastolic diameter; LVEF, left ventricular ejection fraction; PASP, pulmonary arterial systolic pressure*.

### Predictors of HALT in BAV and TAV

From the univariate logistical regression, age, body mass index (BMI), Society of Thoracic Surgeons Predicted Risk of Mortality (STS-PROM), New York Heart Association (NYHA) functional class III/IV, use of anticoagulation, aortic paravalvular leak of ≥ moderate, access, bioprosthetic valve type and D-dimer entered the multivariable logistical regression modeling (Supplementary Table 2). The multivariable logistical regression demonstrated that the anticoagulation and age were independent predictors of both BAV and TAV groups at 1-year (Supplementary Table 3). We didn't find any predictors in the BAV group in 30 days analysis. Transfemoral access and high BMI were protective factors for HALT in the TAV group at 30 days and 1-year, respectively.

### Clinical Events

There was no death during the follow-up, including all-cause and cardiovascular mortality in all groups (Table 6). Four cases (3 in BAV and 1 in TAV) had strokes and one case in BAV had a myocardial infarction. Rehospitalization for any reason was comparable in all four groups (Table 5). There was no statistical difference in the NYHA functional class III/IV, bleeding, and new fibrillation/flutter between the HALT and no HALT groups both in the BAV and TAV during the follow-up. In laboratory tests, D-dimer, and N-terminal pro-brain natriuretic peptide (NT-pro-BNP) were not associated with the HALT both in the BAV and TAV. No matter in which group, there was a strong correlation between the HALT and use of anticoagulation at 1-year, but not at 30 days.

**Table 6:** Clinical outcomes in 30 days and 1-year.

	**BAV**						**TAV**					
	**HALT in** **30 days** ***n =* 25**	**No HALT in** **30 days** ***n =* 140**	***P*-value**	**HALT in** **1-year** ***n =* 52**	**No HALT in** **1-year** ***n =* 97**	***P*-value**	**HALT in** **30 days** ***n =* 19**	**No HALT in** **30 days** ***n =* 172**	***P*-value**	**HALT in** **1-year** ***n =* 62**	**No HALT in** **1-year** ***n =* 104**	***P*-value**
All-cause mortality	0 (0)	0 (0)	–	0 (0)	0 (0)	–	0 (0)	0 (0)	–	0 (0)	0 (0)	–
Cardiovascular mortality	0 (0)	0 (0)	–	0 (0)	0 (0)	–	0 (0)	0 (0)	–	0 (0)	0 (0)	–
All stroke	0 (0)	1 (0.7)	1.000	1 (1.9)	1 (1.0)	1.000	0 (0)	1 (0.6)	1.000	0 (0)	0 (0)	–
Disabling stroke	0 (0)	1 (0.7)	1.000	1 (1.9)	0 (0)	0.349	0 (0)	1 (0.6)	1.000	0 (0)	0 (0)	–
Rehospitalization	1 (5.0)	10 (7.1)	0.723	8 (15.4)	17 (17.5)	0.739	0 (0)	10 (5.8)	0.602	7 (11.3)	13 (12.5)	0.817
Myocardial infarction	0 (0)	1 (0.7)	1.000	0 (0)	0 (0)	–	0 (0)	0 (0)	–	0 (0)	0 (0)	–
Valve endocarditis	0 (0)	0 (0)	–	0 (0)	0 (0)	–	0 (0)	0 (0)	–	0 (0)	0 (0)	–
NYHA functional class III/IV	7 (35.0)	35 (25.0)	0.342	5 (9.6)	9 (9.3)	0.946	6 (31.6)	54 (31.4)	0.987	9 (14.5)	19 (18.3)	0.532
Bleeding	0 (0)	4 (2.9)	1.000	3 (5.8)	4 (4.1)	0.695	0 (0)	2 (1.2)	1.000	2 (3.2)	2 (1.9)	0.630
Major bleeding	0 (0)	1 (0.7)	1.000	2(3.8)	1 (1.0)	0.279	0 (0)	1 (0.6)	1.000	0 (0)	1 (1.0)	1.000
New fibrillation/flutter	0 (0)	2 (1.4)	1.000	1 (1.9)	0 (0)	0.349	0 (0)	1 (0.6)	1.000	0 (0)	3 (2.9)	0.294
D-dimer, ug/L	1575.0 ± 1421.3	1489.1 ± 2092.2	0.203	864.1 ± 560.0	832.7 ± 1058.3	0.844	1053.2 ± 1027.4	1538.7 ± 2050.1	0.324	1093.6 ± 1010.1	844.7 ± 738.1	0.080
NT-ProBNP, pg/ml	868.3 ± 777.4	1190.4 ± 1301.7	0.295	608.4 ± 707.0	725.1 ± 1207.6	0.530	1801.7 ± 1767.0	2218.5 ± 5284.8	0.685	846.6 ± 1026.9	834.2 ± 1433.6	0.954
Use of anticoagulation^*^	3 (15.0)	29 (20.7)	0.767	7 (13.5)	31 (32.0)	0.014	6 (31.6)	45 (26.2)	0.613	9 (14.5)	35 (33.7)	0.007
Warfarin	3 (15.0)	29 (20.7)	0.767	5 (9.6)	31 (32.0)	0.002	6 (31.6)	45 (26.2)	0.613	7 (11.3)	34 (32.7)	0.002
Rivaroxaban	0 (0)	0 (0)	–	2 (3.8)	0 (0)	0.120	0 (0)	0 (0)	-	2 (3.2)	1 (1.0)	0.556

*Values are mean ± SD or number (%)*.

* *Number was counted at the day of pre-CT procedure*.

*BAV, bicuspid aortic valve; HALT, hypoattenuated leaflet thickening; NT-ProBNP, N-terminal pro-brain natriuretic peptide; NYHA, New York Heart Association; TAV, tricuspid aortic valve*.

## Discussion

This study demonstrated that (1) subclinical leaflet thrombosis in BAV and TAV patients was comparable within 30 days or at 1-year; (2) It seemed that the EOA of bioprothesis was different between the HALT and non-HALT group at 1-year follow-up; (3) use of anticoagulation and age were independent predictors both in BAV and TAV; (4) relevant clinical events were similar between the HALT and no HALT groups in BAV and TAV groups.

As the TAVR has been frequently performed in younger and lower-risk patients, the durability of the bioprosthetic valves became a concern in the past years. The SLT was an important cause of bioprosthetic valve dysfunction and compromised the durability of bioprosthetic valves (12). Fortunately, the SLT could be treated and reversed by anticoagulants in many cases (1, 5). Therefore, it may be important to diagnose and treat the SLT to maintain the durability of bioprosthetic valves. At present, some studies have evaluated the leaflet thrombosis of bioprosthetic valves in the TAVR and SAVR procedures, which showed no difference in leaflet thrombosis between the two groups at 1 year (6, 7). Except for durability, Szilveszter et al. found that the SLT was associated with impaired reverse remodeling of left ventricle after the TAVR (13).

Among those younger and lower risk AS patients, the BAV accounted for a large proportion due to the earlier onset in BAV patients. Besides, severe and asymmetric BAV stenosis had some anatomical variations, such as heavily calcified leaflet and the presence of raphe (14), which might have caused under-expansion and malformation of the TAVR stent frame. Those characteristics have increased the concern about leaflet thrombosis and durability in BAV. Waksman et al. found 10.2% HALT from 61 low-risk BAV patients with TAVR at 30 days (8). In this study, the results regarding HALT in BAV patients within 30 days were in line with that previous study. Besides, we explored the differences of HALT between the BAV and TAV groups at early and medium-term follow-up.

In this study, the occurrence of HALT was similar to the outcomes of the prior studies (3, 5, 6). However, we found no difference in HALT between BAV and TAV at early-term (within 30 days) or medium-term (1 year) follow-up. Those anticipated effects of SLT didn't appear to play a role.

In line with the previous studies (15, 16), the MAG value in the HALT or no HALT group was comparable in 1-year follow-up. However, we found that the EOA in the HALT group was smaller than in the no HALT group at 1-year. Of note, MAG in the HALT group was higher than in the no HALT group, although the difference was not significant (HALT vs. no HALT; 11.5 ± 5.6 vs. 10.6 ± 5.1, *p* = 0.164). As is well-known, there was a high correlation between the EOA and MAG. But the difference of the EOA between the HALT and no HALT group might be enlarged by the square calculation. Therefore, it was reasonable to suppose that the MAG might be significantly higher in the HALT group with a longer follow-up.

Except for the age, the use of anticoagulants was an independent predictor for HALT, regardless of in BAV or TAV. The GALILEO-4D study demonstrated that rivaroxaban reduced the risk of RELM in TAVR patients significantly (5). However, this phenomenon wasn't observed within 30 days in this present study. There might be two reasons: (1) almost all patients who needed anticoagulation received warfarin, which required some time to reach a targeted international normalized ratio (INR); (2) more than 90% of the patients completed first CT scan before discharge so that anticoagulation might not have worked at all. We found the transfemoral access was a protective factor for HALT in the TAV group at 30 days. There was a possible reason involved. Almost patients of non-transfemoral access were received the transapical access, which might affect myocardial contractility due to the surgical trauma during the perioperative period. Low ejection fraction of left ventricle was associated with high occurrence of HALT (2). In this study, we also found that the high BMI was associated with the low occurrence of HALT in the TAV group at 1-year follow-up. We didn't know the nature behind this phenomenon. In previous studies, Abhishek Sharma et al. found patients with higher BMI had better outcomes after TAVR (17). In addition, the aortic paravalvular leak may have been a potential protective factor on SLT (Table 5; Supplementary Table 3), which could have changed the hemodynamic status near the bioprosthesis. This result needs to be confirmed by the studies with the larger sample size.

In previous studies, resolution or regression of the HALT/RELM was observed in half of the patients with HALT from 30 days to 1-year follow-up (6, 7). The rate of resolution or regression of the HALT/RELM was low in the present study. A possible reason was that higher occurrence of HALT/RELM at 30-day follow-up was observed in their studies. However, almost all patients in this study completed the first CT scan before discharge. Lars Sondergaard et al. found regression was more likely to be observed if the first CT scan was obtained at >3months after TAVR (3).

In this study, only a few clinical adverse events were observed. There was no difference between the HALT and no HALT groups in BAV or TAV involvement. Some studies showed a higher rate of stroke, transient ischemic attack (TIA), and thromboembolic complications or stroke, and the TIAs were higher in patients with the HALT than in patients with no HALT (2, 6). However, the relationship between the SLT and clinical adverse events still needs a larger sample size trials to confirm.

There were some limitations in this study. First, this was a retrospective study that couldn't avoid some bias, for example, selective bias. Second, the time point of the CT scan was not the exact timepoint of the SLT occurrence. Third, the sample size was not large enough to assess the differences of clinical adverse events.

## Conclusion

The presence of subclinical leaflet thrombosis defined by the CT was comparable between the BAV and TAV in the first year after the TAVR procedure. Age and anticoagulation were the independent predictors of the subclinical leaflet thrombosis at 1 year after the TAVR.

## Data Availability Statement

The raw data supporting the conclusions of this article will be made available by the authors, without undue reservation.

## Ethics Statement

The studies involving human participants were reviewed and approved by the Medical Ethics Committee of the Second Affiliated Hospital Zhejiang University School of Medicine. Written informed consent for participation was not required for this study in accordance with the national legislation and the institutional requirements.

## Author Contributions

GZ: collection, analysis and explanation of data, and drafting of the manuscript. JF: collection, analysis and explanation of data, and revising of the manuscript. DZ, HD, QZ, YH, and YG: collection and analysis of data. XL: conception, design, and revising of the manuscript. JW: conception. All authors contributed to the article and approved the submitted version.

## Funding

This work was supported by Zhejiang Province Science and Technology Department Key R&D Program (2021C03097).

## Conflict of Interest

The authors declare that the research was conducted in the absence of any commercial or financial relationships that could be construed as a potential conflict of interest.

## Publisher's Note

All claims expressed in this article are solely those of the authors and do not necessarily represent those of their affiliated organizations, or those of the publisher, the editors and the reviewers. Any product that may be evaluated in this article, or claim that may be made by its manufacturer, is not guaranteed or endorsed by the publisher.
