# Supplementary material for: Subclinical Leaflets Thrombosis After Transcatheter Replacement of Bicuspid vs. Tricuspid Aortic Valve
Source: Front Cardiovasc Med. 2021 Dec 22;8:790069. doi: 10.3389/fcvm.2021.790069 (PMC8727537; doi:10.3389/fcvm.2021.790069)
Supplement: Supplementary file 1 [file Data_Sheet_1.docx]

| **Supplemental TABLE 1a** HALT in SEV Subgroup | | | |  |
| --- | --- | --- | --- | --- |
|  | **BAV**  n=150 | **TAV**  n=164 | p value | |
| Age, yrs | 74.9±6.6 | 76.6±6.8 | 0.023 | |
| Male | 86 (57.3) | 97 (59.1) | 0.745 | |
| BMI, kg/m^2^ | 22.5±3.2 | 22.6±3.6 | 0.850 | |
| STS PROM, % | 6.0±3.7 | 6.9±5.1 | 0.067 | |
| Smoking | 23 (15.3) | 27 (16.5) | 0.785 | |
| Hypertension | 81 (54.0) | 99 (60.4) | 0.255 | |
| Diabetes mellitus | 35 (23.3) | 33 (20.1) | 0.490 | |
| NYHA functional class |  |  |  | |
| I ~ II | 17 (11.3) | 21 (12.8) | 0.690 | |
| III | 75 (50.0) | 70 (42.7) | 0.194 | |
| IV | 58 (38.7) | 73 (44.5) | 0.294 | |
| Access |  |  |  | |
| Transfemoral | 145 (96.7) | 137 (83.5) | <0.001 | |
| Non transfemoral | 5 (3.3) | 27 (16.5) | <0.001 | |
| Bioprosthetic valve size, mm |  |  |  | |
| ≤23 | 25 (16.7) | 27 (16.4) | 0.961 | |
| >23, ≤26 | 96 (64.0) | 69 (42.1) | <0.001 | |
| >26, ≤29 | 23 (15.3) | 55 (33.5) | <0.001 | |
| > 29 | 6 (4.0) | 13 (7.9) | 0.145 | |
| HALT within 30 days | 17 (144) | 18 (155) | 0.959 | |
| HALT at 1 year | 43 (129) | 47 (135) | 0.800 | |
| Values are mean ± SD or number (%) or number (number).  BMI=body mass index; HALT=hypoattenuated leaflet thickening; NYHA=New York Heart Association; PROM=Predicted Risk of Mortality; SEV=Self-Expanding Valve; STS=Society of Thoracic Surgeons | | | |  |

| **Supplemental TABLE 1b** HALT of SEV vs BEV and MEV | | | |  |
| --- | --- | --- | --- | --- |
|  | **SEV**  n=314 | **BEV/MEV**  n=57 | p value | |
| Age, yrs | 75.7±6.7 | 78.1±6.5 | 0.013 | |
| Male | 183 (58.3) | 34 (59.6) | 0.847 | |
| BMI, kg/m^2^ | 22.6±3.5 | 22.0±3.4 | 0.270 | |
| STS PROM, % | 6.4±4.5 | 8.1±4.5 | 0.009 | |
| Smoking | 50 (15.9) | 6 (10.5) | 0.295 | |
| Hypertension | 180 (57.3) | 32 (56.1) | 0.868 | |
| Diabetes mellitus | 68 (21.7) | 14 (24.6) | 0.627 | |
| NYHA functional class |  |  |  | |
| I ~ II | 38 (12.1) | 4 (7.0) | 0.265 | |
| III | 145 (46.2) | 23 (40.4) | 0.416 | |
| IV | 131 (41.7) | 30 (52.6) | 0.216 | |
| Access |  |  |  | |
| Transfemoral | 282 (89.8) | 57 (100.0) | 0.008 | |
| Non transfemoral | 32 10.2) | 0 (0.0) | 0.008 | |
| Bioprosthetic valve size, mm |  |  |  | |
| ≤23 | 52 (16.6) | 19 (33.3) | 0.781 | |
| >23, ≤26 | 165 (52.5) | 26 (45.6) | 0.335 | |
| >26, ≤29 | 78 (24.8) | 12 (21.0) | 0.539 | |
| > 29 | 19 (6.1) | 0 (0.0) | 0.054 | |
| HALT within 30 days | 35 (299) | 4 (52) | 0.395 | |
| HALT at 1 year | 90 (264) | 24 (51) | 0.078 | |
| Values are mean ± SD or number (%) or number (number).  BEV=Balloon-Expandable Valve; BMI=Body Mass Index; HALT=HypoAttenuated Leaflet Thickening; MEV=Mechanically Expanding Valve; NYHA=New York Heart Association; PROM=Predicted Risk of Mortality; SEV=Self-Expanding Valve; STS=Society of Thoracic Surgeons | | | |  |

| **Supplemental TABLE 2** Univariable Predictors of HALT | | | | | | | | | |
| --- | --- | --- | --- | --- | --- | --- | --- | --- | --- |
|  | **BAV** | | | |  | **TAV** | | | |
|  | OR in 30 days  (95% CI) | p value | OR at 1-year  (95% CI) | p value |  | OR in 30 days  (95% CI) | p value | OR at 1-year  (95% CI) | p value |
| Age, yrs | 1.107 (0.994, 1.160) | 0.070 | 1.064 (1.006, 1.125) | 0.030 |  | - | 0.303 | 1.046 (0.995, 1.099) | 0.076 |
| Female | - | 0.809 | - | 0.283 |  | - | 0.709 | - | 0.367 |
| BMI, kg/m2 | - | 0.538 | - | 0.814 |  | - | 0.290 | 0.913 (0.834, 0.999) | 0.047 |
| STS PROM, % | - | 0.311 | - | 0.234 |  | 0.878 (0.758, 1.018) | 0.084 | - | 0.109 |
| Smoking | - | 0.551 | - | 0.740 |  | - | 0.938 | - | 0.549 |
| Dyslipidemia | - | 0.188 | - | 0.797 |  | - | 0.677 | - | 0.415 |
| Hypertension | - | 0.281 | - | 0.282 |  | - | 0.828 | - | 0.330 |
| Diabetes mellitus | - | 0.159 | - | 0.211 |  | - | 0.204 | - | 0.499 |
| NYHA functional class III/IV | 2.455 (0.939, 6.413) | 0.067 | - | 0.975 |  | - | 0.987 | - | 0.532 |
| Use of anticoagulation* | - | 0.550 | 0.264 (0.108, 0.646) | 0.004 |  | - | 0.613 | 0.355 (0.156, 0.806) | 0.013 |
| Mean aortic gradient | - | 0.920 | - | 0.721 |  | - | 0.388 | - | 0.108 |
| Aortic paravalvular leak ≥ moderate | - | 0.155 | 0.208 (0.046, 0.949) | 0.043 |  | - | 0.337 | 0.261 (0.056, 1.211) | 0.086 |
| Mitral regurgitation ≥ moderate | - | 0.155 | - | 0.557 |  | - | 0.606 | - | 0.558 |
| LVEF, % | - | 0.477 | - | 0.671 |  | - | 0.106 | - | 0.678 |
| Access** | - | 0.391 | - | 0.521 |  | 3.319 (1.139, 9.672) | 0.028 | - | 0.405 |
| Bioprosthetic valve size ≤ 26mm | - | 0.870 | - | 0.362 |  | - | 0.120 | - | 0.155 |
| Bioprosthetic valve type |  |  |  |  |  |  |  |  |  |
| Self-expanding valve | - | - | - | - |  | - | - | - | - |
| Balloon-expandable valve | - | 0.999 | - | 0.242 |  | - | 0.651 | 2.213 (0.920, 5.322) | 0.076 |
| Mechanically expanding valve | - | 0.114 | - | 0.307 |  | - | 0.373 | 0.749 (0.140, 4.009) | 0.736 |
| Postdilation | - | 0.361 | - | 0403 |  | - | 0.519 | - | 0.553 |
| NT-ProBNP |  | 0.295 |  | 0.530 |  |  | 0.685 |  | 0.954 |
| D-dimer |  | 0.203 |  | 0.844 |  |  | 0.324 | 1.000(1.000,1.001) | 0.080 |
| Values are mean ± SD or number (%). *number was counted at the day of pre ct procedure. **Access included transfemoral and non-transfemoral.  BAV=bicuspid aortic valve; BMI=body mass index; HALT=hypoattenuated leaflet thickening; LVEF=left ventricular ejection fraction; NYHA=New York Heart Association; OR=odds ratio; PROM=Predicted Risk of Mortality; STS=Society of Thoracic Surgeons | | | | | | | | | |

| **Supplemental TABLE 3** Multivariable Predictors of HALT | | | | | | | | | |
| --- | --- | --- | --- | --- | --- | --- | --- | --- | --- |
|  | **BAV** | | | |  | **TAV** | | | |
|  | OR in 30 days  (95% CI) | p value | OR at 1-year  (95% CI) | p value |  | OR in 30 days  (95% CI) | p value | OR at 1-year  (95% CI) | p value |
| Age, yrs | - | 0.068 | 1.070 (1.007, 1.136) | 0.028 |  | - | - | 1.057 (1.001, 1.117) | 0.046 |
| BMI, kg/m^2^ | - | - | - | - |  | - | - | 0.908 (0.828, 0.997) | 0.043 |
| STS PROM, % | - | - | - | - |  | - | 0.070 | - | - |
| NYHA functional class III/IV | - | 0.061 | - | - |  | - | - | - | - |
| Use of anticoagulation* | - | - | 0.261 (0.102, 0.665) | 0.005 |  | - | - | 0.370 (0.153, 0.894) | 0.027 |
| Aortic paravalvular leak ≥ moderate | - | - | - | 0.056 |  | - | - | - | 0.103 |
| Access** | - | - | - | - |  | 3.319 (1.139, 9.672) | 0.028 | - | - |
| Bioprosthetic valve type |  |  |  |  |  |  |  |  |  |
| Self-expanding valve | - | - | - | - |  | - | - | - | - |
| Balloon-expandable valve | - | - | - | - |  | - | - | - | 0.307 |
| Mechanically expanding valve | - | - | - | - |  | - | - | - | 0.218 |
| D-dimer | - | - | - | - |  | - | - | - | 0.857 |
| Values are mean ± SD or number (%). *number was counted at the day of pre ct procedure. **Access included transfemoral and non-transfemoral.  BAV=bicuspid aortic valve; BMI=body mass index; HALT=hypoattenuated leaflet thickening; NYHA=New York Heart Association; OR=odds ratio; PROM=Predicted Risk of Mortality; STS=Society of Thoracic Surgeons | | | | | | | | | |
